# HuR binding to AU-rich elements present in the 3' untranslated region of *Classical swine fever virus*

**DOI:** 10.1186/1743-422X-8-340

**Published:** 2011-07-06

**Authors:** Muthukumar Nadar, Meng-Yu Chan, Shi-Wei Huang, Chin-Cheng Huang, Joseph T Tseng, Ching-Hsiu Tsai

**Affiliations:** 1 Graduate Institute of Biotechnology, National Chung Hsing University, Taichung, 402, Taiwan; 2 Department of Hog Cholera, Animal Health Research Institute, Council of Agriculture, Taipei, Taiwan; 3 Institute of Bioinformatics, National Cheng Kung University, Tainan 701, Taiwan

## Abstract

**Background:**

*Classical swine fever virus *(CSFV) is the member of the genus *Pestivirus *under the family *Flaviviridae*. The 5' untranslated region (UTR) of CSFV contains the IRES, which is a highly structured element that recruits the translation machinery. The 3' UTR is usually the recognition site of the viral replicase to initiate minus-strand RNA synthesis. Adenosine-uridine rich elements (ARE) are instability determinants present in the 3' UTR of short-lived mRNAs. However, the presence of AREs in the 3' UTR of CSFV conserved in all known strains has never been reported. This study inspects a possible role of the ARE in the 3' UTR of CSFV.

**Results:**

Using RNA pull-down and LC/MS/MS assays, this study identified at least 32 possible host factors derived from the cytoplasmic extracts of PK-15 cells that bind to the CSFV 3' UTR, one of which is HuR. HuR is known to bind the AREs and protect the mRNA from degradation. Using recombinant GST-HuR, this study demonstrates that HuR binds to the ARE present in the 3' UTR of CSFV *in vitro *and that the binding ability is conserved in strains irrespective of virulence.

**Conclusions:**

This study identified one of the CSFV 3' UTR binding proteins HuR is specifically binding to in the ARE region.

## Background

**Figure 1:**
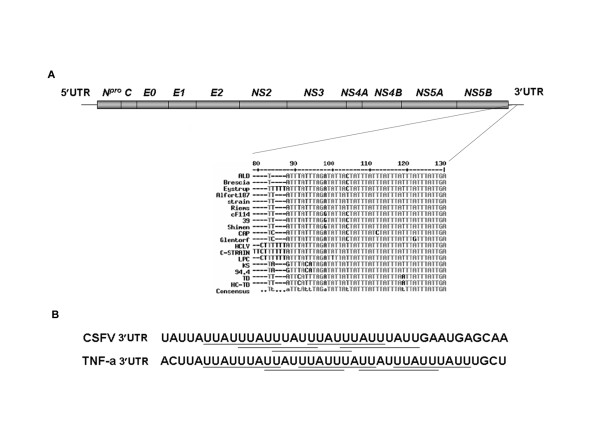
**The illustration of the sequence of adenosine-uridine rich elements present in the 3'UTR of CSFV**. (A) Genome organization of CSFV. The conserved AREs present in the 3' UTR of various CSFV strains are shown. (B) CSFV ARE compared with a type II ARE present in the 3' UTR of tumor necrosis factor alpha (TNF-α) mRNA. The overlapping ARE nonamers are underlined.

*Classical swine fever virus *(CSFV), along with *Bovine viral diarrhea virus *(BVDV) and *Border disease virus *(BDV), is a member of the genus *Pestivirus *under the family *Flaviviridae*. CSFV infects pigs and causes high economic losses especially in Europe and Southeast Asia. CSFV infection causes lymphopenia and thrombocytopenia, thereby evading and compromising the immune system [1]. CSFV is an enveloped icosahedral virus containing a positive sense RNA genome that is devoid of a 5' cap or a 3' poly(A) tail. The 5' untranslated region (UTR) comprises an internal ribosomal entry site (IRES) that drives the translation of a single open reading frame (ORF; Figure 1A) and encoding a polyprotein approximately 4,000 amino acids long, which is co- and post-translationally processed by viral and host proteases into individual structural and non-structural proteins [2]. Due to the limitation of its genome size and the number and functions of encoded proteins, the virus exploits the cellular machinery and components to complete its lifecycle. Cellular host factors could either aid in or interfere with the various stages of the virus lifecycle participating in diverse functions, such as translation, replication, countering host defense, and so on [3-6]. Host factors could either bind to the viral proteins or to the viral RNA.

The 5' UTR of CSFV contains the IRES, which is a highly structured element that recruits the translation machinery. The 3' UTR is usually the recognition site of the viral replicase to initiate minus-strand RNA synthesis [7,8]. The 3' UTR is also known to influence translation, either by interacting with the 5' UTR or by other means [9]. Adenosine-uridine rich elements (ARE) containing mRNA are considered highly unstable, and the translation of which is necessary only for a short period, as in the case of transcription factors, cytokines, and oncogenes [10], and in certain RNA transcripts of DNA viruses [11]. Apart from mRNAs, AREs are also found in non-coding RNAs of viruses [12-14]. Cellular proteins, such as Tristetraprolin (TTP), ARE/poly(U)-binding/degradation factor 1 (AUF 1), Hu antigen R (HuR), and T-cell internal antigen-1 (TIA-1), bind to cellular mRNAs that contain AREs. Upon binding, the ARE binding proteins (AREBPs) mostly target the mRNA to the degradation pathway, for instance, for TTP and AUF1 [15,16], or protect the mRNA from degradation, for example, with HuR [17], or suppress translation, as is the case with HuR and TIA [18,19].

AU-rich sequences have previously been reported to be present in the 3' UTR of all known strains [20,21]. This study found that this AU-rich sequence that CSFV harbors at its 3' UTR is indeed a typical AU-rich element classified as type II ARE, which contains at least two AUUUA pentamer motifs in a U-rich region [10]. This ARE is conserved in all strains of CSFV. Using CSFV 3' UTR as bait, we isolated the interacting host factors and identified several of them employing LC/MS/MS. HuR is one of the proteins identified. This study found via EMSA that HuR binds to the 3' UTR of CSFV RNA *in vitro*. In addition, we also found that HuR binding ability is independent of strain virulence.

## Methods

### Constructs

Plasmids templates for T7 *in vitro *transcription containing the 3'UTR of CSFV strains ALD, LPC, 93KS, and 95TD are derived from the partial cDNA clone of each strain. In brief, the primers used in the reaction are the 5' primer CSFV/T7+12,058 (5'TAATACGACTCACTATAGGGTATGAGCGCGGGTAACCCGGGATCTGGA3'), and the 3' primers ALD3'12328 (5'GGGCCGTTAGGAAATTACCTTAGTC3') and LPC3'12269 (5'GGGCCGTTAGAAATTACCTTA3') for ALD and LPC, respectively. For 93KS and 95TD clones, the 5' primers are CSFV/T7+93KS (5'TAATACGACTCACTATAGGGCACATGAGTGCGGGTAG3') and CSFV/T7+95TD (5'TAATACGACTCACTATAGGGTGTGAGAACGGCCGGCCC3') with the common 3' primer used for ALD strain, ALD3'12328. The T7 promoter sequence is underlined. PCR was performed using the kinased primers and *pfu *DNA polymerase. The insert was ligated into *Sma*I digested and dephosphorylated pUC18 vector; and the positive clones were verified by sequencing. These clones were designated as pT7-ALD3'UTR, pT7-LPC3'UTR, pT7-93KS3'UTR and pT7-95TD 3'UTR.

### RNA labeling

A typical 10 μl *in vitro *transcription reaction for RNA body-labeling contains 2 μl 5X T7 buffer [200 mM Tris-HCl pH-8.0, 40 mM MgCl_2_, 10 mM spermidine-(HCl)_3 _and 125 mM NaCl], 1 μl of 100 mM Dithiotheratol, 20 units T7 RNA polymerase, 1 μg linearized template, 0.25 μl containing 25 mM each of ATP, CTP, and GTP, 0.25 μl containing 250 μM UTP, 3.5 μl of [α-^32^P]UTP containing a total of 11.65 pmole (35 μCi) and 20 units HPRI (Human placental RNAse inhibitor). The reaction was scaled up proportionally as required. Plasmids pT7-ALD3'UTR, pT7-LPC3'UTR, pT7-93KS 3'UTR and pT7-95TD 3'UTR DNA clones were linearized using *Sma*I for run-off transcription RNA labeling. The transcription reaction was performed at 37°C for 2 hrs and the samples were run on a 5% polyacrylamide gel. The appropriate bands were cut and the RNA was eluted from the gel. The ^32^P body-labeled RNA was checked for quality and then quantified.

### Electrophoretic mobility shift assay (EMSA)

Binding was performed in a 10 μl reaction containing 1 μl of 10X binding buffer, 100 ng of yeast total RNAs, about 1 picomole of radio-labeled probe and 1 μg of protein (either GST or GST-HuR, in the case of BSA 2 μg was used ) and 20 units HPRI. For competition assay equal mass (about 5 ng) of cold competitor RNA was used. For super shift assay, 1 μg of anti-HuR antibody was included in the reaction. The reaction was incubated for 10 min at room temperature and then run on a 5% native polyacrylamide gel. The gel was exposed to a phosphorimage plate for a few minutes and then scanned using PhosphorImager Fujifilm BAS 2500.

### PK-15 cytoplasmic extract preparation

PK-15 (Porcine Kidney-15) cells were grown at 37°C in Eagle's minimal essential medium (EMEM) with 5% newborn calf serum (NCS) and 100 U/ml penicillin and 100 μg/ml streptomycin. Sub-cellular fractions were prepared as described [22]. Briefly, PK-15 cells grown in twelve 15 cm^2 ^dishes were washed with PBS, harvested and then washed in 5 volumes of hypotonic buffer and finally suspended in 3 volumes of hypotonic buffer and placed on ice for 10 min. Cells were lysed using a type B pestle Dounce homogenizer by using 10 to 20 strokes on ice. The lysate was centrifuged at 3300 g for 15 min at 4°C and the supernatant was saved as the cytoplasmic extract and its protein content estimated. The pellet contains nuclei.

### RNA-agarose beads cross-linking and protein recovery

In order to be cross-linked, 25 μg of RNA was first oxidized in a 50 μl reaction containing 100 mM sodium acetate pH 5.0 and 5 mM sodium meta-periodate which was incubated for 1.5 hrs at RT in a shaker shielded from light. About 80 μl of agarose beads (Adipic acid dihydrazide Agarose; Sigma) were washed 4 times with and then re-suspended in 60 μl of 100 mM sodium acetate pH 5.0. Oxidized RNA was added to the agarose beads and incubated for 2.5 hr at RT. Then the RNA-agarose beads were washed thrice with 1 ml of 2 M NaCl then washed thrice in buffer D (20 mM HEPES-KOH pH 7.6, 100 mM KCl, 200 mM EDTA, 500 mM DTT, and 5% v/v glycerol).

About 60, 90, and 120 μg of PK-15 cytoplasmic extracts along with 10, 15, and 20 μg yeast total RNAs, respectively, were incubated for 5 min at RT, then added to RNA-agarose beads and placed in a shaker for 25 min at RT (HPRI was added to all reactions at 2 U/μl reaction ratio). The beads were spun down and washed four times in 1 ml of buffer D containing 125 mM NaCl. Then the beads were suspended in 10 μl 4× SDS-PAGE loading buffer and boiled for 5 min and loaded on to a 12% SDS-polyacrylamide gel and run for 65 min at 200V. The gel was stained with silver SNAP and bands of the indicated sizes were cut and sent for mass spectroscopy.

### Plasmids and Purification of GST-HuR Proteins

For constructing the GST-HuR expression vectors, the coding region of HuR was amplified by PCR from human A431 cells cDNA using primers 5'TCTAATGGTTATGAAGACCACATG3' and 5'TTATTTGTGGGACTTGTTGG3', and cloned into pGEX-6P-1 vector (Amersham Bioscience). GST-HuR was prepared using *E. coli *BL21/DE3 cells transformed with pGEX-HuR. GST fusion protein was purified on glutathione-Sepharose beads as recommended by the manufacturer, and the concentration determined by comparison with a bovine serum albumin (BSA) curve in SDS-PAGE stained with Coomassie brilliant blue.

## Results

### CSFV 3' UTR harbors AREs

A detailed analysis revealed five overlapping AUUUA pentamers. This pentamer is a typical adenosine and uridine-rich element (ARE), an instability determinant found in the 3' UTR of short-lived cellular mRNAs, which usually encode proteins required only for a short period, such as transcription factors, oncogenes, and cytokines [10]. AREs are most often found as repeats within U-rich regions. In CSFV 3' UTR, this pentamer is found as five repeats within three UUAUUUAUU tandem-overlapping nonamer repeats (Figure 1B) indicative of type II ARE [10,23]. Type II ARE is typical of mRNAs of cytokines such as TNFα. This ARE repeat is present at approximately 105 nt to 130 nt (nucleotides are numbered from the stop codon) of the 3' UTR of all the known strains of CSFV, and is approximately conserved across these strains, as determined by multiple sequence alignment (Figure 1A). Since viruses usually employ strategies to increase RNA synthesis and evade detection and degradation by host defenses, discovering an instability determinant in the genomic RNA of a virus was intriguing. AREs are the target of a set of proteins that bind to ARE containing RNA collectively, called ARE-binding proteins (AREBPs), which degrade or protect ARE containing RNA or repress its translation upon binding [23].

### Identification of host factors that bind to CSFV 3' UTR

**Figure 2:**
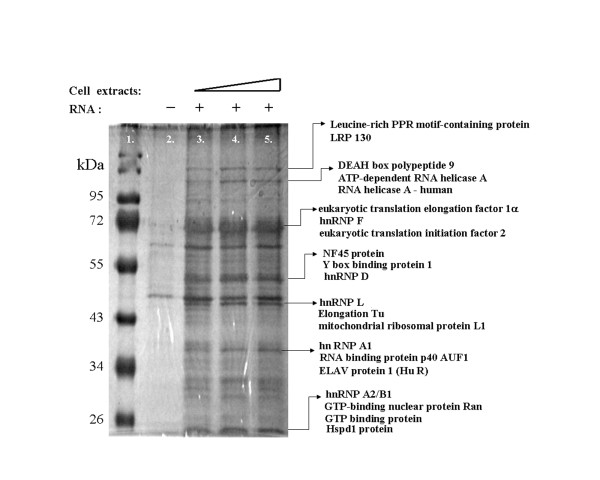
**RNA binding protein isolated from RNA pull-down experiment**. Silver stained SDS-polyacrylamide gel containing the proteins pulled down from PK-15 cytoplasmic extracts using agarose beads linked to CSFV ALD 3' UTR. Lane 1 is the protein size marker; lane 2 is proteins from only beads without RNA, lane 3-5 shows 60, 90, and 120 μg of PK-15 cytoplasmic extract used in the pull down reaction. Some of the identity of the proteins are shown with arrows to represent the bands identified by LC/MS/MS.

Agarose beads covalently cross-linked to CSFV 3' UTR RNA were used to pull down the proteins binding to the 3' UTR present in cytoplasmic extracts of PK-15 cells [24]. The eluted proteins were resolved on an SDS-gel, and selected bands (Figure 2) were cut and sent for LC/MS/MS. Results revealed approximately 32 proteins listed in Table 1. Three of the proteins identified above hnRNP A1, hnRNP D, and HuR are bona fide AREBPs [23]. This study chose HuR for future experiments due to its function of protecting or at least delaying the onset of ARE RNA degradation [17,25].

**Table 1:** LC/MS/MS results of cellular proteins associating with the CSFV 3'UTR

No	Cellular protein	Mr (kDa)	Coverage*
1	Leucine-rich PPR motif-containing protein	153.2	12%
2	RNA helicase A	142	13%
3	ATP-dependent RNA helicase A isoform 2	141.1	16%
4	Nucleolin	76.6	13%
5	ATP-dependent RNA helicase DDX3X	73.2	12%
6	hnRNP Q	69.6	7%
7	ATP-dependent RNA helicase DDX5X	69.1	6%
8	RNA binding protein 47	64	6%
9	Far upstream element binding protein 3	61.6	7%
10	60 kDa heat shock protein	60.9	6%
11	hnRNP L	60.4	14%
12	RNA binding protein FUS	52.3	5%
13	Eukaryotic translation initiation factor 2	51	11%
14	Eukaryotic translation elongation factor 1A (EF1A)	50	19%
15	hnRNP H	49.2	3%
16	Lupus La protein	46.8	4%
17	hnRNP F	45.6	15%
18	NF45 protein	44.7	29%
19	DNA binding protein A	40	13%
20	hnRNP A2/B1	37.4	27%
21	Mitochondria ribosomal protein L1	36.6	10%
22	ELAV protein 1 (HuR)	36	3%
23	Y box binding protein 1	35.9	9%
24	hnRNP A1	34.2	15%
25	hnRNP D	33.4	16%
26	RNA binding protein p40 AUF1	32.6	25%
27	hnRNP A/B	30.8	16%
28	hnRNP D isoform c	29.6	21%
29	40S ribosomal protein S3	27.7	33%
30	Hspd1 protein	26.9	11%
31	GTP-binding nuclear protein Ran	25.5	23%
32	GTP-binding protein	24.4	31%

* The coverage is used to indicate the percentage of the database protein sequence covered by matching peptides.

### HuR binds to the ARE of CSFV 3' UTR *in vitro*

**Figure 3:**
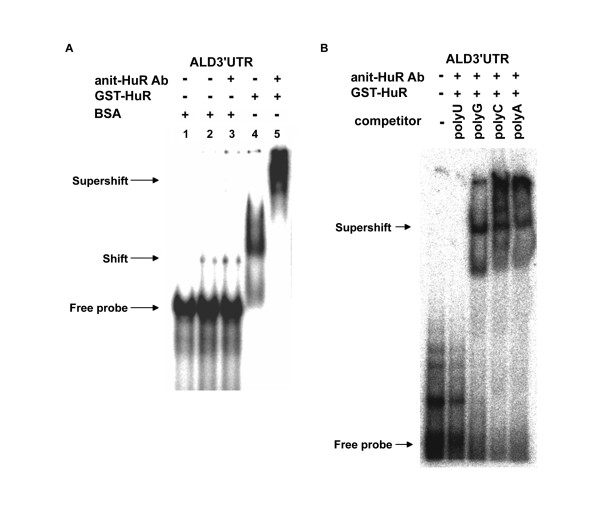
**The specific interaction between HuR and the 3' UTR of CSFV RNA**. GST-HuR binds to CSFV ALD 3' UTR. An autoradiogram of electrophoretic mobility shift assay (EMSA) runs on a 5% non-denaturing PAGE. (A) Free probe (^32^P labeled CSFV ALD3'UTR) without any proteins is loaded in lane 1, 1 μg BSA in lanes 2 and 3, and GST-HuR in lanes 4 and 5 are used in an EMSA. The extra1 μg of anti-HuR antibody is included in lanes 3 and 5. (B) EMSA with unlabeled homo-polyribonucleotides. Free probe without protein components is loaded in lane 1, about 1 μg each of GST-HuR and anti-HuR antibody are loaded with equal mass cold homo-polyribonucleotides, polyU in lane 2, polyG in lane 3, polyC in lane 4, and polyA in lane 5. Arrows indicate the positions of the free, shifted, and super-shifted radiolabeled probes.

To assess whether CSFV 3' UTR binds to HuR, *E. coli*-purified GST-HuR was used in EMSA. Radioactive-labeled CSFV ALD 3' UTR RNA probe (about 1 picomole) interacts with GST-HuR, though not with BSA, even at a higher concentration (Figure 3A). To further confirm this, an antibody against HuR was included in EMSA reaction, resulting in a super-shift only in GST-HuR, and not when incubated with BSA (Figure 3A). This confirms that CSFV 3' UTR binds to HuR *in vitro*. To discover which region of the CSFV 3' UTR binds to HuR, a super-shift competition assay was performed using CSFV 3' UTR as a probe, and equal masses (about 5 ng) of polyA, polyC, polyG, and polyU were used as competitors (Figure 3B). Only polyU was able to out-compete the CSFV 3' UTR (Figure 3B), which is an indirect indication that HuR binds to the ARE of the CSFV 3' UTR, since the ARE is rich in uridines compared to any other region of the CSFV 3' UTR.

### ARE conserved across all strains of CSFV retains *in vitro *HuR binding ability

**Figure 4:**
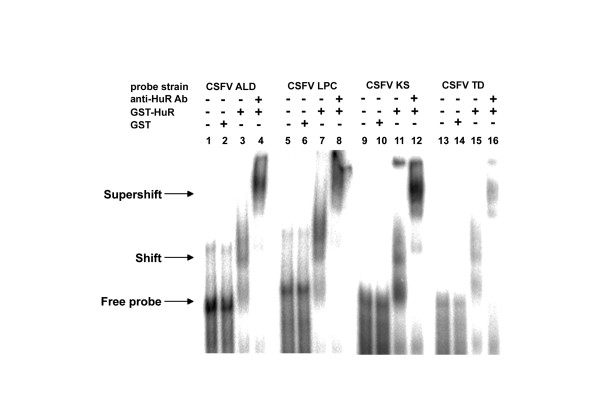
**The interaction of HuR with the 3' UTR conserved in CSFV strains irrespective of virulence**. The 3' UTR of CSFV strains of varied virulence bind to GST-HuR *in vitro*. Autoradiogram of EMSA of the ^32^P labeled RNA of the 3' UTR belonging to four different CSFV strains ALD, LPC, 93KS, and 96TD run on a 5% non-denaturing polyacrylamide gel. Free probe derived from each strain is loaded in lanes 1, 5, 9, and 13. The purified GST only as the control is loaded in lanes 2, 6, 10, and 14; and GST-HuR as the binding partner (lanes 3, 7, 11, and 15) and anti-HuR antibody are included in the super-shift reactions (lanes 4, 8, 12, and 16) indicated above the lanes. Arrows indicate the positions of the free, shifted, and super-shifted radiolabeled probes.

CSFV comprises numerous strains of varied virulence and infectivity that cause diseases of varied severity, ranging from acute to sub-acute and chronic symptoms. They all possess the ARE in their 3' UTR, irrespective of virulence. Even the avirulent vaccine strain LPC contains this sequence in addition to the polypyrimidine stretch that precedes the ARE. This study sought to discover if HuR could bind to the 3' UTR of CSFV strains irrespective of virulence, and chose four strains, ALD (a highly virulent strain), 93KS and 96TD (both moderately virulent Taiwanese isolates), and LPC (an avirulent vaccine strain).^32^P-labeled 3' UTR of the aforementioned strains were used in EMSA containing purified GST or purified GST-HuR (Figure 4). The anti-HuR antibody was then included in the EMSA super-shift reaction containing GST-HuR. Results show a shift in mobility in reactions containing purified GST-HuR compared to the free probe or the negative control that contains purified GST only (Figure 4). In reactions that contain the anti-HuR antibody in addition to GST-HuR, a clear super-shift is observed, indicating that HuR binds to the ARE of CSFV 3' UTR, irrespective of its nature of infection.

## Discussion

We have demonstrated that CSFV harbors bona fide AREs in the 3' UTR of its genomic RNA and that it is conserved in all analyzed strains. To the best of our knowledge, this is the first report showing that a viral genomic RNA of a positive-sense RNA animal virus harbors ARE motifs in its 3' UTR. The presence of an instability determinant such as the ARE in the genomic RNA of positive-sense RNA virus is seemingly suicidal. The presence of ARE in cellular mRNAs or certain RNAs of specific DNA viruses is understandable, though its presence in the sole RNA of CSFV appears harmful to its existence. However, since CSFV is extremely efficient in infectivity and multiplication and has a broad tissue tropism, the presence of the ARE hints that the virus could use the ARE interacting machinery of the host to its advantage.

Using RNA agarose beads, we isolated host factors from PK-15 cytoplasmic extracts that interact with the CSFV 3' UTR, and identified many of them using LC/MS/MS. Data attained via LC/MS/MS indicate more than one AREBP; we chose to focus on HuR first because it has been reported to protect ARE containing mRNAs from degradation. The coverage of HuR is only 3%, which could be because HuR is a nuclear/cytoplasmic protein, though it is predominantly nuclear. This study has shown that purified recombinant GST-HuR binds to CSFV 3' UTR *in vitro *(Figures 3 and 4).

Though HuR is predominantly nuclear, various types of stress, such as UV exposure, chemical stress, and heat shock, cause HuR to relocalize to the cytoplasm [26-29]. While CSFV genomic RNA is restricted to the cytoplasm and HuR is predominantly nuclear, viral infection is a type of stress and, as the name implies, the typical symptom of CSFV infection is high fever, which is a heat shock stress. In addition, reports show that HuR shuttles between the nucleus and the cytoplasm at various stages of the cell cycle. HuR is predominantly cytoplasmic during S-phase to M-phase transition [30], whereas in T cells, HuR is predominantly cytoplasmic during the early G1 phase [26]. The facts stated above hint at a possibility that when HuR relocalizes to the cytoplasm during such conditions, it might play a role in the CSFV lifecycle. The binding of HuR to CSFV 3' UTR and the repression of translation [19,31] may be the trigger that switches the process of translation to RNA replication.

The advent of low molecular weight inhibitors of HuR, which have demonstrated its drugability, could open exciting possibilities into the functional and mechanistic studies of HuR, and also in the therapeutic potential of combating viral infections [32]. The role of HuR in the CSFV lifecycle, and the interaction of other AREBPs with the CSFV 3' UTR ARE and their functions, is the scope of our future study.

## Conclusions

In conclusion, this study used the RNA pull-down and proteomics techniques to identify several host proteins interacting with the 3' UTR of CSFV RNA. One of the CSFV 3' UTR binding proteins, HuR specifically binds to the ARE region in the 3' UTR.

## Competing interests

The authors declare that they have no competing interests.

## Authors' contributions

MN prepared the HuR protein and did the EMSA analysis. MYC did the RNA pull down experiments. SWH prepare the labeled transcripts. CCH provided critical review of the manuscript. JTT and CHT were project supervisors, participated in the discussion of all experiments of the project and preparation of the manuscript.

All authors read and approved the manuscript

## References

[B1] Sanchez-Cordon PJ Nunez A Salguero FJ Pedrera M Fernandez de Marco M Gomez-Villamandos JC Lymphocyte apoptosis and thrombocytopenia in spleen during classical swine fever: role of macrophages and cytokines Vet Pathol 2005 42 477 488 10.1354/vp.42-4-477 16006607

[B2] Kolupaeva VG Pestova TV Hellen CU Ribosomal binding to the internal ribosomal entry site of classical swine fever virus RNA 2000 6 1791 1807 10.1017/S1355838200000662 11142379 PMC1370049

[B3] Ahlquist P Noueiry AO Lee WM Kushner DB Dye BT Host factors in positive-strand RNA virus genome replication J Virol 2003 77 8181 8186 10.1128/JVI.77.15.8181-8186.2003 12857886 PMC165243

[B4] Bushell M Sarnow P Hijacking the translation apparatus by RNA viruses J Cell Biol 2002 158 395 399 10.1083/jcb.200205044 12163463 PMC2173839

[B5] Gale M Jr Foy EM Evasion of intracellular host defence by hepatitis C virus Nature 2005 436 939 945 10.1038/nature04078 16107833

[B6] Lin JW Ding MP Hsu YH Tsai CH Chloroplast phosphoglycerate kinase, a gluconeogenetic enzyme, is required for efficient accumulation of Bamboo mosaic virus Nucleic Acids Res 2007 35 424 432 17169994 10.1093/nar/gkl1061 PMC1802604

[B7] Cheng JH Peng CW Hsu YH Tsai CH The synthesis of minus-strand RNA of bamboo mosaic potexvirus initiates from multiple sites within the poly(A) tail J Virol 2002 76 6114 6120 10.1128/JVI.76.12.6114-6120.2002 12021344 PMC136226

[B8] Xiao M Chen J Wang Y Zhen Y Lu W Li B Sequence, necessary for initiating RNA synthesis, in the 3'-noncoding region of the classical swine fever virus genome Mol Biol 2004 38 343 351 15125241

[B9] Ito T Tahara SM Lai MM The 3'-untranslated region of hepatitis C virus RNA enhances translation from an internal ribosomal entry site J Virol 1998 72 8789 8796 9765423 10.1128/jvi.72.11.8789-8796.1998 PMC110295

[B10] Chen CY Shyu AB AU-rich elements: characterization and importance in mRNA degradation Trends Biochem Sci 1995 20 465 470 10.1016/S0968-0004(00)89102-1 8578590

[B11] Tan W Schwartz S The Rev protein of human immunodeficiency virus type 1 counteracts the effect of an AU-rich negative element in the human papillomavirus type 1 late 3' untranslated region J Virol 1995 69 2932 2945 7707519 10.1128/jvi.69.5.2932-2945.1995 PMC188992

[B12] Cook HL Mischo HE Steitz JA The Herpesvirus saimiri small nuclear RNAs recruit AU-rich element-binding proteins but do not alter host AU-rich element-containing mRNA levels in virally transformed T cells Mol Cell Biol 2004 24 4522 4533 10.1128/MCB.24.10.4522-4533.2004 15121869 PMC400482

[B13] Iseni F Garcin D Nishio M Kedersha N Anderson P Kolakofsky D Sendai virus trailer RNA binds TIAR, a cellular protein involved in virus-induced apoptosis EMBO J 2002 21 5141 5150 10.1093/emboj/cdf513 12356730 PMC129035

[B14] Li W Li Y Kedersha N Anderson P Emara M Swiderek KM Moreno GT Brinton MA Cell proteins TIA-1 and TIAR interact with the 3' stem-loop of the West Nile virus complementary minus-strand RNA and facilitate virus replication J Virol 2002 76 11989 12000 10.1128/JVI.76.23.11989-12000.2002 12414941 PMC136884

[B15] DeMaria CT Brewer G AUF1 binding affinity to A+U-rich elements correlates with rapid mRNA degradation J Biol Chem 1996 271 12179 12184 10.1074/jbc.271.21.12179 8647811

[B16] Lai WS Carballo E Strum JR Kennington EA Phillips RS Blackshear PJ Evidence that tristetraprolin binds to AU-rich elements and promotes the deadenylation and destabilization of tumor necrosis factor alpha mRNA Mol Cell Biol 1999 19 4311 4323 10330172 10.1128/mcb.19.6.4311 PMC104391

[B17] Peng SS Chen CY Xu N Shyu AB RNA stabilization by the AU-rich element binding protein, HuR, an ELAV protein EMBO J 1998 17 3461 3470 10.1093/emboj/17.12.3461 9628881 PMC1170682

[B18] Piecyk M Wax S Beck AR Kedersha N Gupta M Maritim B Chen S Gueydan C Kruys V Streuli M Anderson P TIA-1 is a translational silencer that selectively regulates the expression of TNF-alpha EMBO J 2000 19 4154 4163 10.1093/emboj/19.15.4154 10921895 PMC306595

[B19] Yeh CH Hung LY Hsu C Le SY Lee PT Liao WL Lin YT Chang WC Tseng JT RNA-binding protein HuR interacts with thrombomodulin 5'untranslated region and represses internal ribosome entry site-mediated translation under IL-1 beta treatment Mol Biol Cell 2008 19 3812 3822 10.1091/mbc.E07-09-0962 18579691 PMC2526687

[B20] Vilcek S Belak S Organization and diversity of the 3'-noncoding region of classical swine fever virus genome Virus Genes 1997 15 181 186 10.1023/A:1007971110065 9421882

[B21] Vilcek S Paton D Lowings P Bjorklund H Nettleton P Belak S Genetic analysis of pestiviruses at the 3' end of the genome Virus Genes 1999 18 107 114 10.1023/A:1008000231604 10403696

[B22] Abmayr SM Yao T Parmely T Workman JL Preparation of nuclear and cytoplasmic extracts from mammalian cells Curr Protoc Mol Biol 2006 Chapter 12 Unit 12 11 10.1002/0471142727.mb1201s75 18265374

[B23] Barreau C Paillard L Osborne HB AU-rich elements and associated factors: are there unifying principles? Nucleic Acids Res 2005 33 7138 7150 10.1093/nar/gki1012 16391004 PMC1325018

[B24] Langland JO Pettiford SM Jacobs BL Nucleic acid affinity chromatography: preparation and characterization of double-stranded RNA agarose Protein Expr Purif 1995 6 25 32 10.1006/prep.1995.1004 7538839

[B25] Dean JL Wait R Mahtani KR Sully G Clark AR Saklatvala J The 3' untranslated region of tumor necrosis factor alpha mRNA is a target of the mRNA-stabilizing factor HuR Mol Cell Biol 2001 21 721 730 10.1128/MCB.21.3.721-730.2001 11154260 PMC86664

[B26] Atasoy U Watson J Patel D Keene JD ELAV protein HuA (HuR) can redistribute between nucleus and cytoplasm and is upregulated during serum stimulation and T cell activation J Cell Sci 1998 111 3145 3156 9763509 10.1242/jcs.111.21.3145

[B27] Bandyopadhyay S Sengupta TK Spicer EK PMA induces stabilization of oncostatin M mRNA in human lymphoma U937 cells Biochem J 2008 410 177 186 10.1042/BJ20070311 17924856

[B28] Gallouzi IE Brennan CM Stenberg MG Swanson MS Eversole A Maizels N Steitz JA HuR binding to cytoplasmic mRNA is perturbed by heat shock Proc Natl Acad Sci USA 2000 97 3073 3078 10.1073/pnas.97.7.3073 10737787 PMC16194

[B29] Mazan-Mamczarz K Galban S Lopez de Silanes I Martindale JL Atasoy U Keene JD Gorospe M RNA-binding protein HuR enhances p53 translation in response to ultraviolet light irradiation Proc Natl Acad Sci USA 2003 100 8354 8359 10.1073/pnas.1432104100 12821781 PMC166233

[B30] Kim HH Gorospe M Phosphorylated HuR shuttles in cycles Cell Cycle 2008 7 3124 3126 10.4161/cc.7.20.6884 18927508 PMC2577782

[B31] Kullmann M Gopfert U Siewe B Hengst L ELAV/Hu proteins inhibit p27 translation via an IRES element in the p27 5'UTR Genes Dev 2002 16 3087 3099 10.1101/gad.248902 12464637 PMC187493

[B32] Meisner NC Hintersteiner M Mueller K Bauer R Seifert JM Naegeli HU Ottl J Oberer L Guenat C Moss S Identification and mechanistic characterization of low-molecular-weight inhibitors for HuR Nat Chem Biol 2007 3 508 515 10.1038/nchembio.2007.14 17632515

